# Barriers and Facilitators to Participation in Clinical Trials Related to Familial Frontotemporal Dementia: A Qualitative Study

**DOI:** 10.1002/mgg3.70038

**Published:** 2024-11-25

**Authors:** David Zammitt, Emilie V. Brotherhood, Caroline Fearn, Caroline Greaves, Ollie Hayes, Emma Harding, Madalena Lykourgos, Jonathan D. Rohrer, Josh Stott

**Affiliations:** ^1^ ADAPT Lab, Department of Clinical Educational and Health Psychology University College London London UK; ^2^ Dementia Research Centre University College London London UK; ^3^ Department of Clinical Educational and Health Psychology University College London London UK

**Keywords:** clinical trials, familial, frontotemporal dementia, preclinical, recruitment

## Abstract

**Aims:**

Familial frontotemporal dementia (fFTD) is an inherited neurodegenerative condition characterised by executive dysfunction, impairments in social cognition, behaviour and language. Although no disease‐modifying interventions are currently available, several treatments are undergoing clinical trials. This study sought to understand the barriers and facilitators to taking part in such trials, as well as general perceptions of the treatments undergoing trial.

**Method:**

Twelve interviews took place with fourteen participants: eight individuals who were genetically at‐risk of developing fFTD, two individuals diagnosed with fFTD and four spousal carers. Their views and experiences of clinical trials were explored using thematic analysis.

**Results:**

Five main themes were developed: (1) effects on the individual, (2) implications for others, (3) systemic considerations, (4) the impact of genetic status and disease progression and (5) the role of communication and understanding.

**Conclusions:**

The decision to participate in clinical trials was said to be complex, involving consideration of logistical barriers alongside health implications. Participants identified potential advantages of participating in clinical trials to be direct health benefits and the ability to help others, however risks to participants and their families' physical and psychological wellbeing were also named. Relationships between organisations and participants were consistently considered to be important, with lack of psychological care at various timepoints post diagnosis; unclear communication and expectation‐setting; and inadequate organisational collaboration all identified as barriers. Participants indicated that increased health‐professional interest in FTD and an associated increase in opportunities would be key facilitators for greater participation in clinical trials.

## 1 Introduction

Frontotemporal dementia (FTD) is an umbrella term used to describe a group of neurodegenerative conditions associated with frontal and/or temporal lobar atrophy (Sivasathiaseelan et al. 2019). FTD comprises several subtypes including: behavioural variant (bvFTD) (Gambogi et al. 2021), associated with progressive change in personality and behaviour as well as executive dysfunction (Johnen and Bertoux 2019; da Silva et al. 2021) and the primary progressive aphasias (PPA) (Ghosh and Lippa 2015), characterised by language impairments, including speech production and comprehension (Rascovsky et al. 2011).

FTD comprises around 5%–10% of all dementia cases (Leroy et al. 2021). However, it is a leading cause of dementia in individuals under 60 (Seo et al. 2018). Age of onset is variable, even among individuals from the same family and generation (Moore et al. 2020). The burden of care associated with FTD is high, with research suggesting that the per‐patient economic cost of FTD may be substantially higher than that associated with Alzheimer's disease (Galvin et al. 2017).

Although FTD can develop sporadically, it has been shown to be highly heritable, with approximately 30%–50% of cases linked to a clear family history (Greaves and Rohrer 2019; Onyike and Diehl‐Schmid 2013). Inherited cases of FTD are referred to as familial FTD (fFTD) (Russell 2022) and autosomal dominant mutations in three genes have been found to account for the majority of such heritability: progranulin (GRN), microtubule‐associated protein tau (MAPT) and chromosome 9 open reading frame 72 (C9orf72) (Greaves and Rohrer 2019). Although no disease‐modifying treatments are currently available for any form of FTD (Sivasathiaseelan et al. 2019), multiple biomarkers associated with fFTD have been identified, and several treatments aimed at targeting these are undergoing trial (Greaves and Rohrer 2019). In this context, clinical trials can be a source of support and hope for individuals at risk of developing fFTD. However, in line with broader research on dementia (Cooper, Ketley, and Livingston 2014) and on Huntington's disease (HD) (Carroll et al. 2022), another heritable and neurodegenerative condition; a relatively small proportion of eligible individuals enter these studies.

To our knowledge, no prior research has explored the reasons underlying low uptake of clinical trials among this population. We hypothesise that the requirement to undergo testing for presence of the genetic mutation associated with fFTD may be a factor because 70%–80% of individuals identified as at risk of developing fFTD do not pursue genetic testing (Greaves and Rohrer 2019). Other factors (e.g. emotional, organisational and practical) may also influence decision‐making, as well as elements which researchers have not yet considered. For example, wider dementia research indicates suggests trials involving a substantial degree of physical risk or a high level of personal burden (Nuño et al. 2017). Research on HD also suggests that individuals may choose not to be genetically tested due to the inability to unlearn the information after receiving it, as well as the absence of effective treatments (Quaid and Morris 1993). Those who find out that they carry associated genetic mutations have cited a negative impact upon mental health (Hagberg, Bui, and Winnberg 2011). It is possible that such reasons may also contribute to poor levels of participation in clinical trials related to fFTD, and people with preclinical FTD are more anxious than their unaffected family members (Cheran et al. 2018).

Outside of dementia, many obstacles to participation in clinical trials have been found (Rodríguez‐Torres, González‐Pérez, and Díaz‐Pérez 2021; National Institutes of Health 2015; Sheridan et al. 2020) and these may also be an issue in fFTD.

FTD has profound effects on affected individuals' lives, and the associated burden of care is substantial. With recent research indicating that slowing the progression of other forms of dementia may be possible (Lilly 2023), achieving greater trial participation may facilitate specific progress in relation to fFTD. To realise this aim, the views and experiences of this population must be explored, including individuals at risk of fFTD, those who have developed the condition, and their caregivers. The current study aimed to gain an understanding of the barriers and facilitators to taking part in such trials, as well as more general perceptions of the treatments undergoing trial, including related procedures and participation criteria.

## 2 Methods

### 2.1 Ethics

The study was conducted within the scope of NHS ethical approval previously obtained by the GENetic Frontotemporal Initiative study (GENFI; reference number: 14/0377).

### 2.2 Recruitment and Participants

Participants were recruited from the UCL GENFI study cohort (see “Genetic FTD Initiative”), which includes individuals who are either at risk or have a confirmed diagnosis of fFTD and carers. All individuals over the age of 18 who had either been identified as genetically at‐risk of developing fFTD or had a confirmed diagnosis, as well as individuals caring for them, were eligible for this study. Purposive sampling ensured that the perspectives of those who had, and had not, undergone genetic testing as well as those who had, and had not, taken part in clinical trials were all captured. Due to a lack of translation resources, only individuals who could speak English to a fluent level were approached.

### 2.3 Procedure

Those who expressed interest in the study after receiving an initial recruitment email, received further information, including an overview of interview topics. Sample size was guided by a combination of information power (Malterud, Siersma and Guassora, 2016), research on sample size in thematic analysis (Guest, Bunce, and Johnson 2006) and pragmatic considerations, resulting in a desired sample of 10–15 individuals.

Participants were given an opportunity to ask questions before written informed consent was obtained. Semi‐structured individual or dyadic interviews were carried out either over video call (*N* = 11) or telephone (*N* = 1), depending on participant preferences. Interviews ranged from 60 to 90 min in duration and were audio‐recorded.

### 2.4 Interview Schedule

An interview schedule (see Appendix A) was drafted in consultation with clinical experts, including a Consultant Clinical Psychologist, a Consultant Neurologist and two carers of people with FTD. The schedule was developed with attention to any barriers and facilitators to clinical research participation highlighted in previous literature within AD (Nuño et al. 2017) and HD (Hagberg, Bui, and Winnberg 2011; Quaid and Morris 1993) populations, and any additional areas specific to FTD identified by stakeholders. Interviews were semi‐structured to allow opportunity for any additional unanticipated insights.

### 2.5 Researcher Perspective

DZ, who conducted the interviews and analysis, is a White Irish male and was training to be a Clinical Psychologist. He had some clinical experience working with individuals with neurodegenerative conditions but not yet worked with anyone living with FTD. He did not have any personal or familial experience of any form of dementia. He held a fundamentally positive view of clinical trials but attempted to adopt a curious stance in exploration of the topic. Bracketing was employed throughout (e.g. Drew, 2004), which included keeping a reflective journal and having regular supervision sessions with an experienced researcher in the field, as well as consulting two spousal carers of individuals with fFTD as experts by experience.

### 2.6 Analysis

Interviews were transcribed verbatim, anonymised and sent to participants before analysis to ensure that participants felt they were represented accurately, and to give them the opportunity to request deletion of any data they did not want included. Four participants requested minor amendments to transcripts, including the removal of repeated words and informal language.

A reflexive thematic analysis was conducted according to the six stages outlined by Braun and Clarke (2006): familiarisation with the data, generation of initial codes, searching for themes from codes, reviewing themes, defining and naming themes, and writing the report. NVivo software was used (QSR International Pty Ltd. 2020).

As a previously unexplored area in the literature, transcripts were coded inductively, and themes were generated “without trying to fit it into a pre‐existing coding frame or the researcher's analytic preconceptions” (Nowell et al. 2017). The analysis was informed by the core principles of descriptive phenomenology (Sundler et al. 2019), in that we tried to describe, rather than interpret, participants' subjective experiences in a data‐driven manner, whilst “emphasizing openness, questioning pre‐understanding and adopting a reflective attitude” (Sundler et al. 2019, p. 735).

All transcripts were read and re‐read, conducting line‐by‐line coding to develop an initial list of codes. DZ moved back and forth between transcripts several times, applying new codes where relevant. All data were coded, even when not appearing to be related to the study's aims initially, in line with the inductive approach.

### 2.7 Quality Assurance

Appropriate credibility checks for qualitative research in psychology were employed (Elliott, Fischer, and Rennie 1999). First, another trainee clinical psychologist (ML), independently coded two transcripts, before DZ and ML reviewed their respective lists of initial codes, discussed any discrepancies and incorporated these into a joint list. DZ then applied any additional codes where appropriate to the remaining transcripts. Respondent validation (Barker and Pistrang 2005) was also used, to promote a collaborative approach to the analysis, and ensure the representativeness of the study's results. An initial summary of themes, sub‐themes and example quotations was disseminated to all participants in April 2023 for feedback. Four participants responded and their feedback informed the selection of further example quotations which captured a broader representation of participant views.

## 3 Results

### 3.1 Participants

In total, fourteen participants were recruited: eight individuals who were designated as genetically at risk of developing fFTD; two individuals with diagnoses of fFTD; and four spousal carers. Two carers took part in interviews on their own, representing the views of their loved one with FTD who did not take part. Two carers took part in joint (dyadic) interviews, alongside the individuals for whom they cared Participant 8 (person living with frontotemporal dementia, PLWFTD) and Participant 9 (spousal carer); Participant 11 (PLWFTD) and Participant 12 (spousal carer). As such, 12 separate interviews took place.

Of the eight individuals at risk of fFTD, seven of these had received positive genetic testing outcomes. One participant interviewed individually had previously taken part in a clinical trial. The two carers who were interviewed by themselves cared for someone who had taken part in trials. (see Table 1). The mean age of spousal carers was 65 (all female), the mean age of PLWFTD was 70 (both male) and the mean age of participants at risk of fFTD was 46 (half female, half male). All participants identified their ethnicity as White (of British, English, Welsh or Mixed European heritage).

**TABLE 1:** Participant information.

Participant ID	Category	Undertaken genetic testing?	Previously taken part in trials?
1	Spousal carer	N/A	N/A
2	At risk	Yes	No
3	Spousal carer	N/A	N/A
4	At risk	Yes	No
5	At risk	Yes	No
6	At risk	Yes	No
7	At risk	Yes	No
8	PLWFTD	Yes	No
9	Spousal carer of Participant 8	N/A	N/A
10	At risk	No	No
11	PLWFTD	Yes	No
12	Spousal carer of Participant 11	N/A	N/A
13	At risk	Yes	No
14	At risk	Yes	Yes

Abbreviations: N/A, Not applicable; PLWFTD, person living with frontotemporal dementia.

### 3.2 Thematic Analysis

Five themes and eight sub‐themes were generated from the data. An overview of all themes and sub‐themes, and their representation across participants, can be seen within Table 2. An example extract from a transcript where coding has been applied can be found in Appendix B, and an example of the codes which were grouped together to form a single sub‐theme can be found in Appendix C.

**TABLE 2:** Representation of themes and sub‐themes by participant.

Themes		P1	P2	P3	P4	P5	P6	P7	P8	P9	P10	P11	P12	P13	P14
1	*Effects on the individual*	X	X	X	X	X	X	X	X	X	X	X	X	X	X
1.1	Effects on health and FTD symptomatology	X	X	X	X	X	X	X	X	X	X	X	X	X	X
1.2	Effects on mental health and wellbeing	X	X	X	X		X	X		X	X		X	X	X
2	*Implications for others*	X	X	X	X	X	X	X		X	X		X	X	X
3	*Systemic considerations*	X	X	X	X	X	X	X	X	X	X	X	X	X	X
3.1	Logistical hurdles	X	X	X	X	X	X	X	X	X	X	X	X	X	X
3.2	Relationships between participants and organisations	X	X	X	X	X		X		X	X		X	X	X
3.3	Need for increased interest in FTD	X	X	X	X	X	X	X			X		X	X	X
4	*The impact of genetic status and disease progression*	X	X	X	X	X	X	X	X	X	X		X	X	X
5	*The role of communication and understanding*	X	X	X	X	X	X	X	X	X	X		X	X	X
5.1	The importance of good communication	X	X		X	X	X	X	X	X	X		X	X	X
5.2	Setting expectations	X	X		X			X	X	X			X	X	X
5.3	Decoding scientific information		X	X	X		X	X			X		X		X

### 3.3 Effects on the Individual

The decision to take part in clinical trials involved considering the effects of trials upon individuals' physical health, FTD symptomatology and psychological wellbeing. Although there was a sense that the potential benefits of participation outweighed risks, participants highlighted dangers inherent in novel treatments.

#### 3.3.1 Effects on Health and FTD Symptomatology

All participants described potential physical health impacts. For many, this was related to the uncertainty associated with new treatments.

“They're not approved medications … Stage one, where they look to see if there's any harm done … I'd be hesitant to be involved at that sort of point.” Participant 10

Although they generally thought risk of death was low due to robust processes, multiple participants said that it factored into their decision‐making.

“There have been one or two [deaths] over the years … these things can go wrong.” Participant 12

Several participants also voiced concerns around potential treatment delivery methods and other trial procedures (e.g. lumbar punctures) which may result in pain, discomfort or fatigue.

“Well, essentially I was off work for about 10 days after that … I'm happy to do all this stuff, but lumbar puncture is—I would rather avoid it if I can.” Participant 5

Despite this, participants suggested that such concerns would generally not prevent participation, often due to belief that trials may represent the best opportunity to receive effective treatment.

“There isn't going to be another one, so aren't you going to risk it and take it? They can give you another pill to cure that side effect.” Participant 7

#### 3.3.2 Effects on Mental Health and Wellbeing

Concerns around mental health, coupled with a perception of inadequate current psychological support were also voiced by several participants.

The potential effect of learning new information during screening in relation to one's condition and anticipated symptoms onset (e.g. their neurofilament light chain level) was highlighted.

When you get down to knowing the time period in which your onset is, I think that's getting a bit more crucial in terms of how it will mess with your head. Participant 7

Several participants mentioned concerns that trial participation may lead to unfounded hopes for positive outcomes.

There is absolutely a risk that you go into this trial thinking this might fix me and then it doesn't. And so the comedown from expectation could be massive. Participant 13

Although participants appeared to accept the need for trial protocols, they said that missing out on treatment, due to being part of control groups or not fulfilling eligibility criteria, may affect their mental health.

If I was going through a trial … and then find out that I've been getting the placebo. That would be quite devastating. Participant 10

However, participants also described psychological benefits of taking part in trials in terms of increased agency.

Giving someone control, whether that be the person that it's actually happening to or a family member, in being able to do something positive about it is really important. Participant 14

Although there was an overall sense that genetic counselling during genetic testing had been positive, this was often where FTD‐related psychological support ended. One participant argued that this likely negatively affects trial participation in the long term.

After you receive those diagnoses, there's nothing. You're just dumped, end of … and then we're saying, ‘can you give us your time, your risk, your everything else to do the trial?’ Participant 14

There was a sense that improved psychological support should be offered throughout a participant's journey.

I think actually people with the genetic result need ongoing support psychologically. And especially around being approached for trials. Participant 4

### 3.4 Implications for Others

The majority (*N* = 12) described how decisions around trial participation involved others. Some participants described navigating complex family dynamics due to their condition's heritability. For example, trial participation might reveal their genetic status, with possible implications for family members.

“They could work out that I have the gene. If they found out that I was on a drug trial, … There are some members of the family who just don't need to know.” Participant 4

Several participants also highlighted potential effects of trials on carers or partners, particularly if the trial participant developed side effects.

“[Name] has had medications changed, altered, and has had two episodes of very severe delirium. We're talking months here … So this is a big concern in my mind.” Participant 9

Trial criteria requiring study partners was also described as a potential barrier. Several participants suggested that individuals living on their own, or without someone to monitor their symptoms, may be unable to take part.

“In effect these drug trials exclude people living on their own.” Participant 4

The consideration of others, however, was also highlighted as a potential facilitator, with several participants describing a hope that their trial participation might help others.

“Having had a very negative diagnosis, the desire to try and do something positive, not just for yourself … it's to help children and then their children and other people.” Participant 1

### 3.5 Systemic Considerations

Every participant discussed logistical hurdles. Many spoke positively about relationships they had developed with the organisations involved in trials, with others describing a need for more work on this. More broadly, there was a sense that increased interest in FTD was required if trials are to make progress.

#### 3.5.1 Logistical Hurdles

The majority of participants discussed that taking part in trials involves overcoming logistical obstacles. These included practical burdens such as travel, time, money, and administrative tasks.

There were key logistical obstacles for many including the need for travel, consequent need for work absences, financial burden (e.g. reimbursement processes) and impact on other commitments (e.g. childcare and work):

“He [PLWFTD] was eligible to stay on the drug as a participant in phase two, but we didn't take that option because going to London every four weeks is actually not that straightforward from where we live.” Participant 3

“And then logistics of childcare … Drop the kids, get them both to school, be on a certain train to get there … I would potentially have had to have reduced my hours at work or something.” Participant 2

Several participants hypothesised that making trials available more locally might facilitate greater participation.

#### 3.5.2 Relationships Between Participants and Organisations

Faith in trials, and willingness to participate was mediated by the confidence that individuals had in professionals working within them and that working with knowledgeable and understanding staff facilitates participation.

“We have a link to [organisation] that's spanned years. It feels like we know them. They know us. They have therefore put something our way that feels relevant.” Participant 12

For some, however, a gap existed between the motivations of professionals and participants. For example, one participant, described a negative experience of FTD research.

“There's no bedside manner associated with it at all. None […] I just travelled for 2 and a half or 3 h, needed the toilet. But that can wait until after you filled this thing in.” Participant 13

Others suggested that research participation felt one‐sided because they did not receive information regarding the outcome of their input.

“One of the things that would help is […] knowing what has happened to the research that we've participated in.” Participant 12

#### 3.5.3 Need for Increased Interest in FTD

Several participants suggested lack of awareness and interest in FTD, relative to other conditions was a barrier to participation. They spoke about improving the general public's awareness and understanding and increasing interest among relevant professionals, and political and pharmaceutical spheres, which might lead to more funding and more clinical research.

Multiple participants felt that dementia was not something which occupied public consciousness to the same extent as conditions like cancer.

“I feel like people who are living with dementia, there's just generally less airtime around. So, you hear less about the research. There's less public awareness.” Participant 1

One participant suggested that this overlapped with a lack of political will around addressing dementia's long‐term impacts as elected officials were motivated by shorter term outcomes.

“We do have a kind of a demographic time bomb that is slowly ticking and it's not like people don't know about it. And the problem is that politicians tend to not be elected on the decisions that are going to bear fruit in 20 or 30 years” Participant 5

Others implied that the rarity of FTD may mean that research is not as economically viable.

“Because it's such a very rare disease, are the drug companies invested enough with this to develop something for this because there's not many people with it?” Participant 10

Finally, although multiple participants expressed their appreciation for staff working in FTD research, there was a feeling that more specialised resources were required.

“I'm struck by how hand‐to‐mouth it feels. And in terms of getting the right people with the right skills feeling that they've got a career and a personal stake in solving these things—it doesn't feel like that's where we are at.” Participant 12

### 3.6 The Impact of Genetic Status and Disease Progression

Participants cited genetic status and disease progression information as trial participation considerations. There was a sense that trials which require evidence of genetic status will miss those who either do not know or wish to know their status. For example, one participant said that an option to take part without learning their status may facilitate participation.

“The risk of getting tested and then finding that I've got the mutation and there's no treatments … I don't know how I'd handle that … I guess I would prefer if there was a trial that came along and said, ‘OK, we'll test you, but we won't tell you the results.’” Participant 10

The practical and legal ramifications associated with genetic testing were also cited as potential barriers. Participants mentioned how a positive result may potentially lead to having to leave work, stop driving or having to inform insurance companies. Conversely, several participants hypothesised that learning they may be close to symptom onset could positively influence participation.

“If you already know that you're 1 to 5, or 1 to 3 years from onset—these drugs take a long time to produce, so you've got to weigh up what the likelihood is of something coming along—if it's not the one you're being offered, how long until the next gene therapy comes along?” Participant 7

Moving forward, multiple participants discussed utility of more trials for presymptomatic individuals as this had been a barrier to their own participation.

“I wasn't qualified for that because I'm—touching wood—not currently showing signs. But I would be up for it if it was open to me.” Participant 13

Related to this, another hope was that trials for a greater number of specific genetic mutations might be developed.

“It's a bit limited at present on C9orf72.” Participant 7

### 3.7 The Role of Communication and Understanding

Communication and understanding were mentioned as important by almost all participants. Clear communication and expectation‐setting prior to, during and after trial participation was indicated as key, particularly for those without a high degree of prior healthcare/science related knowledge.

#### 3.7.1 The Importance of Good Communication

Good communication was identified as crucial for improving trial participation rates. Thirteen participants mentioned this as a factor in their decision‐making. Participants spoke about the manner and format of sharing and the importance of being kept informed about trial progress.

Several participants described positive experiences stating that they had received regular updates with the right amount of information from organisations.

“It's all been good. The seminars, superb. The information that they put across, the amount that's going on. It's wonderful.” Participant 13

Others, however, mentioned the confusing nature of how information can flow to prospective participants. For example, one individual was told by healthcare staff that no FTD‐related trials existed and it was only through their sibling's research that they found out this was incorrect.

“We were actually told by the NHS that there's nothing, there's no medical trials or there is nothing really out there that can support this.” Participant 6

Some participants suggested that communication format, particularly email, meant that at times they had not received enough information to make truly informed decisions.

“Because [NAME] was invited to join one just with an email recently, we made our decision [to decline] on that email alone …” Participant 9

Others wanted to receive information about ongoing or past trial outcomes, to better understand progress at any given time.

“I'd really want to know what had happened during that trial to the symptomatic people. How good was it?” Participant 7

#### 3.7.2 Setting Expectations

Relatedly, participants suggested that setting clear expectations around trials was critical. There was an overall feeling that individuals were prepared to accept the risks of learning difficult information or going through difficult procedures, but that they wanted to be sufficiently prepared for this.

One participant, who had found it difficult to get information on their genetic mutation, spoke positively about a researcher setting realistic expectations regarding the likelihood of them developing FTD, suggesting that this may facilitate participation.

“I asked her outright, ‘What is the percentage?’ Because I know that the problem is the research base isn't huge for the gene […]. But she was much more honest about the fact that I almost certainly will get it.” Participant 4

A carer also highlighted the need for clear expectation‐setting around physical procedures as individuals already experiencing the effects of FTD may find it difficult to appreciate the risks.

“When you have a lumbar puncture, they are really good at explaining to you the risk of a headache, the risk of some leakage of fluid, the risk of something more serious. And I think there is potential for people with forms of dementia to be a bit dismissive of the risks.” Participant 12

#### 3.7.3 Decoding Scientific Information

Related to this, many participants referenced needing to improve their scientific understanding of FTD and clinical trials to make informed participation decisions.

For some, prior expertise or experience of working in healthcare or other scientific areas was helpful in allowing them to navigate such information.

“I've got a science background, so it's not quite so difficult for me, possibly, than others. […] I don't know how somebody with less of a science background would have coped with the information.” Participant 4

Others, however, did not have such knowledge to draw upon, affecting understanding, increasing isolation as well as potentially, reducing the likelihood of participation.

“When the drug companies released their published results … unless you've got a degree in neuroscience, as a patient, you're going to struggle to understand it. And selfishly people want to know, ‘Well, is it helping me? Is it going to help me live?’” Participant 14

## 4 Discussion

The present study was the first to examine the barriers and facilitators to participation in clinical trials related to fFTD from the perspective of those affected. Five main themes were identified: (1) effects on the individual, (2) implications for others, (3) systemic considerations, (4) the impact of genetic status and disease progression and (5) the role of communication and understanding.

Below we connect barriers and facilitators to existing literature in other populations. Key overarching facilitating factors were helping oneself or helping others. This is both in line with AD (Bardach, Holmes, and Jicha 2018) and HD (Carroll et al. 2022) research suggesting that more than 90% of these populations are similarly motivated, while contrasting with general research on clinical trials suggesting extrinsic rewards, such as monetary benefit, are the key incentives (Abdelazeem et al. 2022).

Despite a general willingness to accept them, all participants mentioned the potential physical impact of trial participation, echoing broader (Rodríguez‐Torres, González‐Pérez, and Díaz‐Pérez 2021), and dementia‐specific (Watson et al. 2014) trial literature. Specific risks were also cited in relation to the heritable nature of FTD: requirements around genetic testing and biomarker information could impact upon psychological wellbeing; extending on HD findings (Hagberg, Bui, and Winnberg 2011; Quaid and Morris 1993). This may be particularly true of individuals who carry a genetic mutation for which relevant clinical trials are currently lacking, as was the case for several participants. Information gleaned from undertaking genetic testing in order to participate in trials could have profound practical consequences, including financial and legal implications, as outlined in existing literature (Ascencio‐Carbajal et al. 2021). However, finding out proximity to symptom onset, potentially increased willingness to participate for some participants. This connects to work suggesting that people with AD experiencing greater severity of dementia symptoms may be more motivated to participate (Nuño et al. 2017).

Concerns were also voiced around the potential of finding out that they were in a control group post‐trial, which could negatively affect their mental health, echoing work in other populations (Mills et al. 2006) (Hughes‐Morley et al. 2015), including AD (Ottenhoff et al. 2023). Also, issues were raised surrounding the requirement of and implications for study partners, highlighted both here and in other studies (Black et al. 2018). From AD research we understand that this role is most often occupied by spouses (Cary et al. 2015), raising the possibility that not having a spouse is a barrier to trial participation for those with fFTD.

Logistics were consistently identified as important. Travel time commitments and impacts on employment were described as potential barriers and reducing travel burden is one suggested way to improve participation in AD trials (Ballard, Gwyther, and Edmonds 2010; Karlawish et al. 2008).

As described elsewhere (e.g. Natale et al. 2021), the importance of relationships with researchers and organisations was consistently highlighted; these had the potential to be positive and faciliatory or could highlight differences in motivations and result in a perceived lack of appreciation (e.g. Watson et al. 2014; Rodríguez‐Torres, González‐Pérez, and Díaz‐Pérez 2021). These findings emphasise the need to build relationships and support people affected by FTD when enrolling on trials.

As found generally in dementia studies (Bouranis, Gelmon, and Lindauer 2023), lack of good communication was described as a barrier, with participants suggesting that they had not received enough information about, or were unaware of the existence of, trials. Conversely, as in AD (Ottenhoff et al. 2023) and general trials work (Sood et al. 2009) having expectations managed, being kept informed about trial outcomes, and the meaning of screening test results were facilitators. When making decisions around participation, participants felt a need to become experts on FTD and trial processes themselves, reflecting the experiences of carers of people with FTD who also describe this in response to a perceived lack of professional awareness of FTD, (Tookey et al. 2022).

As has been suggested previously (Greaves and Rohrer 2019), participants felt that improved psychological support after initial genetic testing is needed to facilitate trial participation. Looking ahead, participants emphasised the importance of greater public interest in and awareness of dementia and FTD specifically, as a key factor for progress, concurring with existing literature and policy (Robinson et al. 2018; UK Parliament 2021).

### 4.1 Theoretical and Clinical Implications

Several implications for improving trial participation arise from the present study.

A public health focus on increasing awareness of FTD, genetic testing and clinical trials could increase participation could help support participation. Work in other areas (e.g. cancer) suggests that public attitudes towards trials are generally developed through engagement with the media (Grunfeld et al. 2002; Madsen, Holm, and Riis 2007). Increased funding may also attract more professional and pharmaceutical interest.

The use of open‐label placebos (whereby participants are aware that they are receiving a placebo) and, reducing the likelihood of receiving a placebo are potentially helpful design amendments as in some AD and Cancer trials (Kushnir et al. 2022; Watson et al. 2014). Any such changes, however, should be made in tandem with increased support, particularly through improved communication and psychological care throughout individuals' FTD journeys. Empathy, warmth, approachability, and a sense that participants' contributions are valued by researchers may facilitate as they do in AD (Ottenhoff et al. 2023).

Consulted potential participants and carers on trial material design could support ensure expectation management and accessibility of information, dispel possible myths, and encourage participation. Related to this, improving the accuracy of tests related to FTD symptom onset, a need highlighted by Swift et al. (2021), may also help with accurate expectation‐setting.

In line with AD research, education of local health professionals and continuing work to improve networks and research registries are also likely to be beneficial (e.g. Greaves and Rohrer 2019; Russell 2022). Further integration of FTD research within national calls for dementia research participation and nationwide initiatives like Join Dementia Research may stimulate greater awareness and participation in FTD trials (e.g. Cooper, Ketley, and Livingston 2014; Juaristi and Dening 2016).

Improving the mental wellbeing of this population is a crucial standalone goal, however, if successful could also increase the number of participants who feel ready and able to participate in trials. Promising work on the development of psychological interventions for those at risk of fFTD is underway (Greaves et al. 2022).

Finally, although participants in the present study appeared to hold broadly positive views of trials, positive messaging around trials should continue, to further foster and expand the pool of people who see trials as a potential vehicle for progress, as well as to ensure that individuals who do contribute to research gain a meaningful understanding of how their contributions are utilised.

### 4.2 Strengths and Limitations

The involvement of experts by experience in study design and analysis which may optimise healthcare impacts (Ahuja and Williams 2005), and is in line with UK public health policy (NHS England 2017). However, important views are likely to have been missed in this study due to purposive sampling from an existing FTD research database. This pragmatic approach was adopted due to the rarity of the disease and a desire to verify FTD diagnosis. However, a research database population are likely to hold positive views of scientific research and be able to navigate relevant information and organisations, which might not be true of a wider FTD population. This and the high proportion of participants who had taken part in genetic testing (Greaves and Rohrer 2019) may affect the transferability of findings particularly as genetic testing was identified as a potentially key barrier, and so gaining a better understanding of the views of individuals who have decided against this may warrant further exploration.

Also, despite overall efforts to obtain participants with a mix of trial experience, only one participant at risk of fFTD had taken part in a clinical trial, meaning that the views of the other at‐risk participants were based on either their experiences of trial screening processes or broader FTD research. Additionally, the views of two carers who spoke about family members who had taken part in trials must be understood in the context that their opinions may not fully represent the views or experiences of these individuals.

Similarly, whilst it was felt that dyadic interviews were necessary to facilitate the contributions of people living with FTD, carrying out interviews as spousal dyads will have influenced the data arising from these conversations.

All participants identified as White possibly as a function of recruiting from a registry. (Jin et al. 2023). Future research should aim to recruit and understand the views of individuals from a range of ethnic backgrounds.

## 5 Conclusion

This analysis provides novel insights into the views and experiences of individuals affected by fFTD regarding clinical trials with several barriers and facilitators elucidated. We suggest strategies to enhance participation and suggest that future research should aim to explore the views of participant groups who were not reached by the current study, including individuals from a more representative range of ethnic backgrounds as well as those who have not previously taken part in research, clinical trials or genetic testing associated with fFTD.

## Author Contributions

David Zammitt was responsible for study design, collection, analysis and interpretation of data, writing of the report and in the decision to submit the report for publication. All research activities were overseen by Josh Stott. Caroline Greaves offered advice on the study design and supported with recruitment. Madalena Lykourgos had a role in the analysis and interpretation of data. Emilie V Brotherhood, Caroline Fearn, Ollie Hayes, Emma Harding, Jonathan D Rohrer and Josh Stott were involved in writing the report and the decision to submit the report for publication. All authors read and approved the final version of the manuscript.

## Ethics Statement

The study was conducted within the scope of NHS ethical approval by Queen Square Ethic Committee previously obtained by the GENetic Frontotemporal Initiative study (GENFI; reference number: 14/0377). Interviews were undertaken with the full understanding and written consent of each participant.

## Conflicts of Interest

The authors declare no conflicts of interest.

## Supporting information


Appendix S1.

## Data Availability

The data that support the findings of this study may be available on request from the corresponding author. The data are not publicly available due to privacy or ethical restrictions.
