# Supplementary material for: Barriers and Facilitators to Participation in Clinical Trials Related to Familial Frontotemporal Dementia: A Qualitative Study
Source: Mol Genet Genomic Med. 2024 Nov 25;12(11):e70038. doi: 10.1002/mgg3.70038 (PMC11586684; doi:10.1002/mgg3.70038)
Supplement: Supplementary file 1 — Appendix S1. [file MGG3-12-e70038-s001.docx]

DZ Supplement paper on clinical trials

## Appendix A

### Interview Schedule

1. **Introduction**

*The goal of this study is to understand the views of individuals at risk of frontotemporal dementia about clinical trials aimed at developing treatments for the condition.*

*It is an exciting time in this field of research and one of our aims is to help encourage individuals by communicating the depth of interest and ongoing research by various organisations working towards a positive outcome in the future.*

*We want to understand your experience and your opinions in relation to clinical trials, whether or not you have already taken part or plan to take part in any. In particular, we want to understand factors which encourage participation as well as any perceived barriers which either prevent or reduce the likelihood of individuals taking part. It is hoped that this information will help inform the design of future trials in order to improve participation.*

*The interview will last between 60 and 90 minutes. It will be recorded and the answers will be written up so we can consider in detail all that you have said. This will only be carried out by approved individuals, and anything we write will not include names, or any details which could identify you. This means everything you say is anonymous after it is written up.*

*Your participation is entirely voluntary and you can withdraw from the study at any stage up until the point where the study is submitted for publication.*

*Finally, we appreciate that this is an emotional topic. If, for any reason, you become upset in any way during the interview, please feel free to stop or take a break at any point.*

*Do you consent to proceeding with the interview?*

1. **Consent obtained? Y/N**
2. **Participant background information**- For the purposes of the recording, can you tell me your name?
   - What age are you?
   - Are you an individual with FTD? An individual who is at risk for FTD, or a family/carer of an individual who has the diagnosis or has been designated as at risk?
   - Have you undergone genetic testing related to FTD? If applicable, how long ago did this happen?
   - Have you taken part in any clinical trials related to FTD? If so, can you briefly outline these?
3. **Questions
   Understanding & perceptions of trials**
   - Do you know of any treatments which have undergone or are currently undergoing trial? What do you know about these?

**-** Have you been offered a chance to take part in any of these?

**-** How worthwhile do you perceive these to be (this might be in terms of the person, their family, society, future individuals)? We understand that this might be difficult if you have not taken part.

**-** How effective do you perceive these to be? Again, we understand that this might be difficult if you have not taken part.

**-** Do you perceive any risks to be associated with these? This might be risks to physical and/or mental health. *Prompts: How knowledgeable do you feel you are about treatments and clinical trials? Have you received information about these? Has that information been useful? Could that information be improved? Have there been any practical factors (e.g., staff or organisations you have dealt with) which have either helped you understand these, or which have hindered your understanding of these?*

**Wider context: family involvement in trials**
- Have any of your family members taken part in trials?

**-** If so, what was their experience? *Prompts: Did they tell you anything about the process, either positive or negative? Did the trial have any positive or negative impacts upon them?*

**Questions for participants who *have* taken part in trials**
- What were the reasons which drove your decision to take part?

- Did any factors in particular either directly lead to or encourage your participation?

- Were there any barriers which you had to overcome in order to take part? E.g., work, transport.

- Were genetic testing requirements a factor in your decision to take part?

- Could anything be improved about the experience?

- Have there been any perceived benefits or costs/advantages or disadvantages in taking part?

- Is there a message you would give to individuals who have either chosen not to take part or who are still considering this? / With hindsight, how would you have persuaded yourself or your loved one?

- Would you be prepared to speak to someone who is considering taking part, either participant or study partner, anonymously if you would prefer?

- Do you think anything could be improved about the process in order to improve participation?

- How do trials that require knowing your genetic status to enter them affect your views on predictive genetic testing (for those who have not yet been tested)?

- If you have not yet been tested, would you prefer trials to include all people at-risk? This might mean that you could find out your genetic status accidentally if you had a side-effect of the drug.

- How would you feel about taking a test to find out that you are near to the onset of symptoms in order to take part in a trial?
 *Prompts: What emotions did you feel when you decided to take part? What emotions did you feel during and after taking part? Were your family or friends involved in this decision? How did/do you feel about the staff and organisations involved? How do you feel about the process of taking part?*

**Questions for participants who *have* *not* taken part in trials**
- Firstly, can you briefly describe why you have not taken part in trials?

- Have there been any barriers to your participation in trials?

- If so, can you think of any ways to overcome these?

- If you have actively chosen not to take part in trials, what were the reasons which drove your decision? Could anything encourage you to take part?

- Would you consider taking part in the future?

- If you would like to take part in trials, but have not yet been able to, what are the reasons for this?

- Would genetic testing requirements be a factor in your decision to take part?

- For those who have not yet been tested: How would a trial that required knowing your genetic status to enter it make you change your views on predictive genetic testing?

- If you have not yet been tested, would you prefer trials to include all people at-risk? This might mean that you could find out your genetic status accidentally if you had a side-effect of the drug.

- How would you feel about taking a test to find out that you are near to the onset of symptoms in order to take part in a trial?
 *Prompts: Do you feel trials can be useful? Do you have any worries or fears with regard to taking part? Were your family or friends involved in this decision? How do you imagine the process might be?*

**The future**
- Looking further down the line, what do you feel the future holds for clinical trials?

- What are your hopes for clinical trials?

- What do you see as the future for FTD treatments in general?

- Is there anything you would like to see happen or change in this area?
 *Prompts: Are you hopeful for clinical trials? Do you feel clinical trials will be useful for your generation or for future generations?*

1. **Generic prompts**- You spoke about ____, ____, ____ - can you tell me more about ____?
   - Can you give me an example of that?
   - How did that make you feel?
   - What was it that you liked/disliked about that experience?
   - Is there anything you would change about that experience?
   - Is there anything you would like to add?
2. **Closing the interview**- Thank participant for their contributions and remind participant that, as discussed, they can withdraw from the study at any stage.
   - Ask participant how they are feeling and establish if require psychological support.
   - Remind participant of contact details should they have any further questions or need additional support after leaving.

## Appendix B

### Example of Coding Within a Single Transcript Extract

*Extract without codes applied*


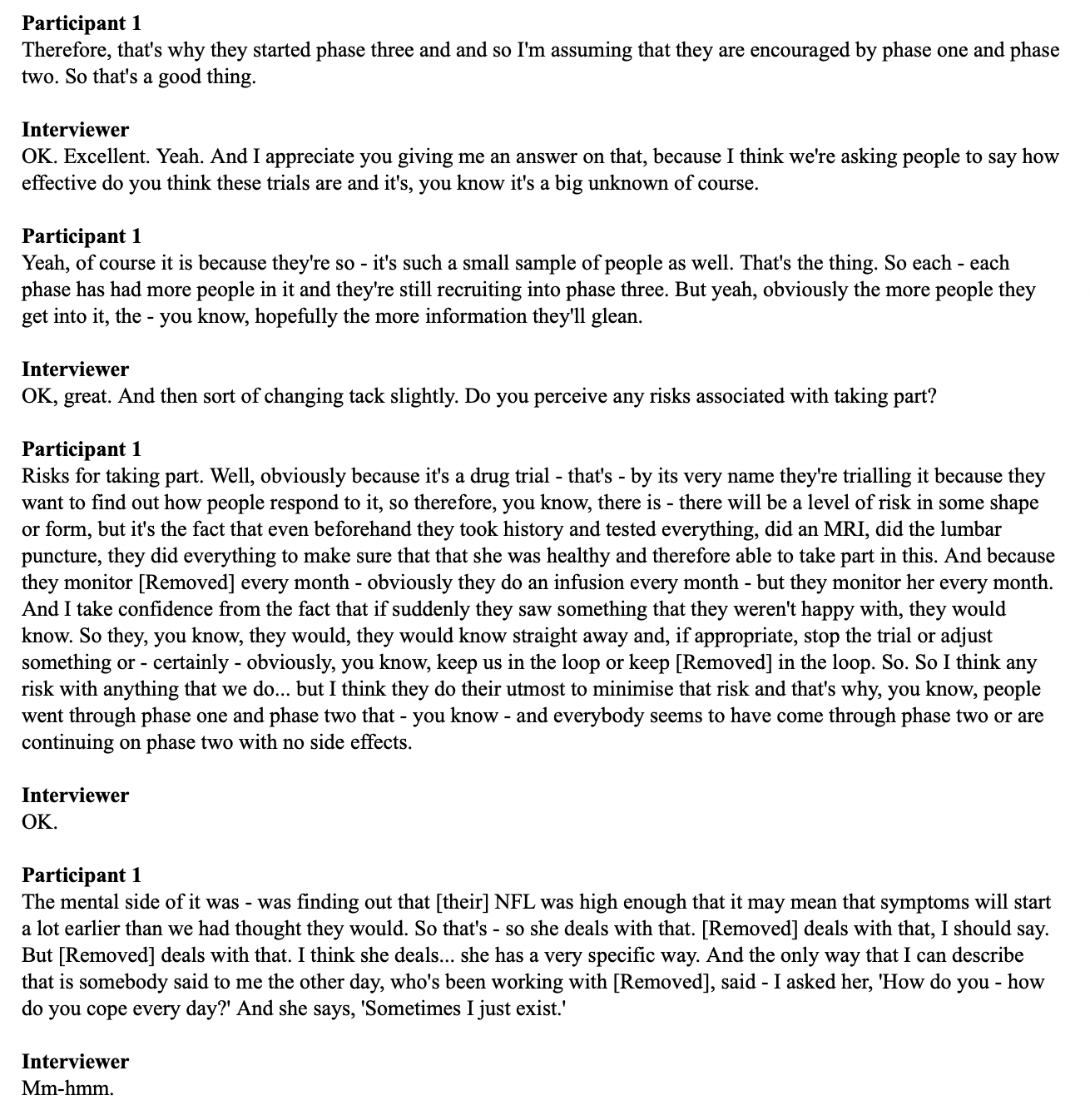


*Code: Faith in professionals*


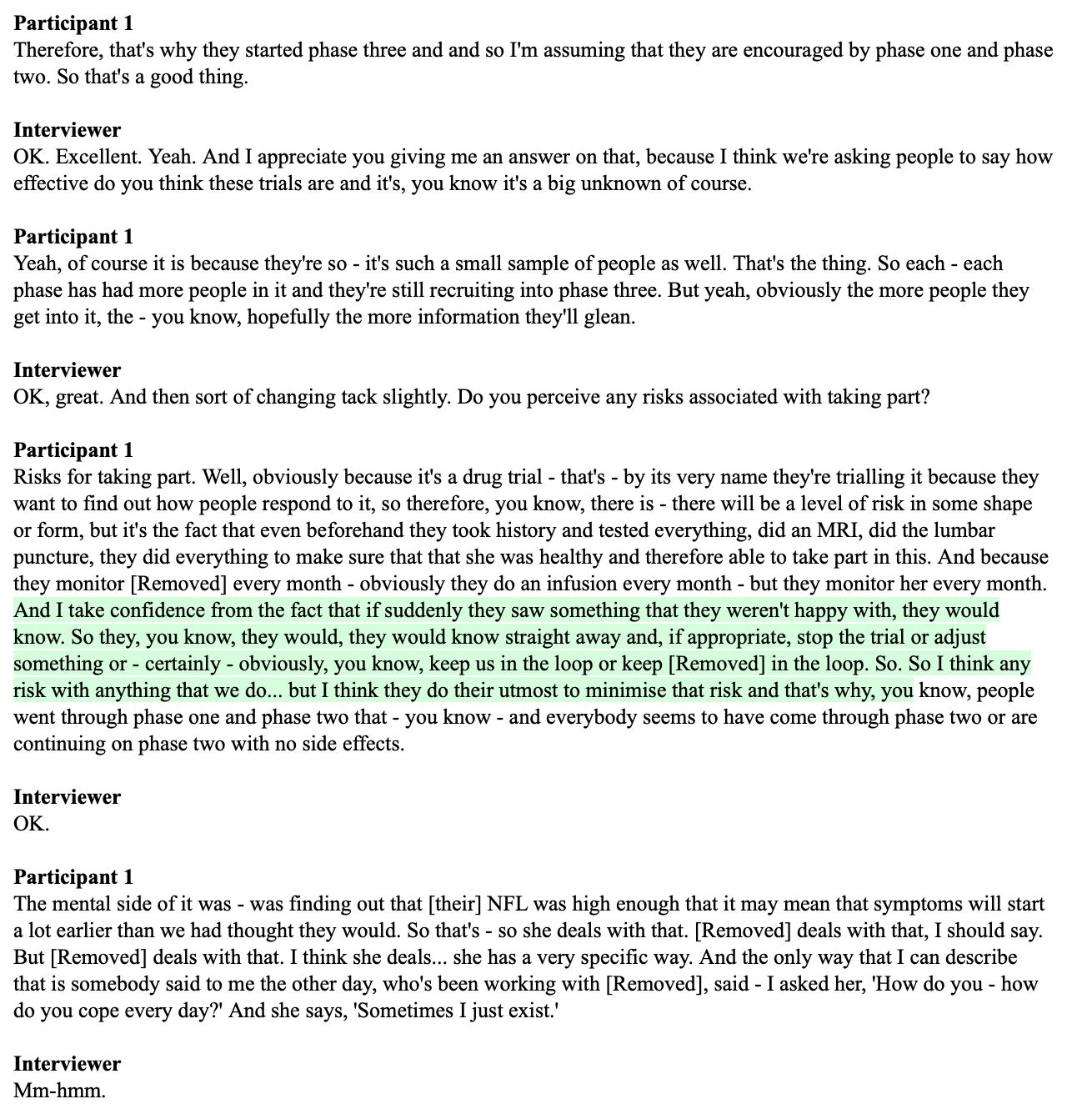


*Code: Perception that trial results are encouraging*

*
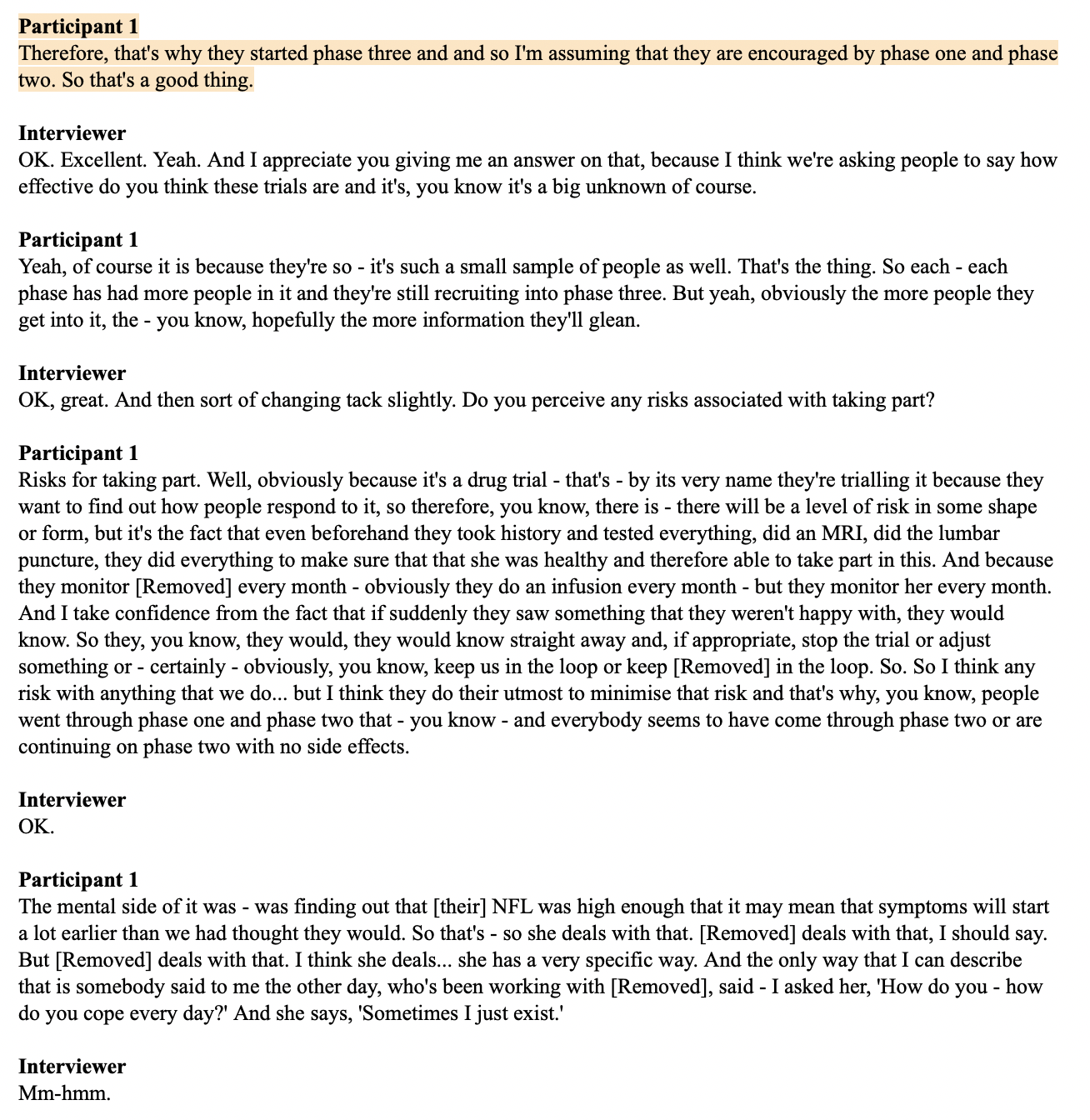
*

*Code: Willingness to accept a degree of risk*


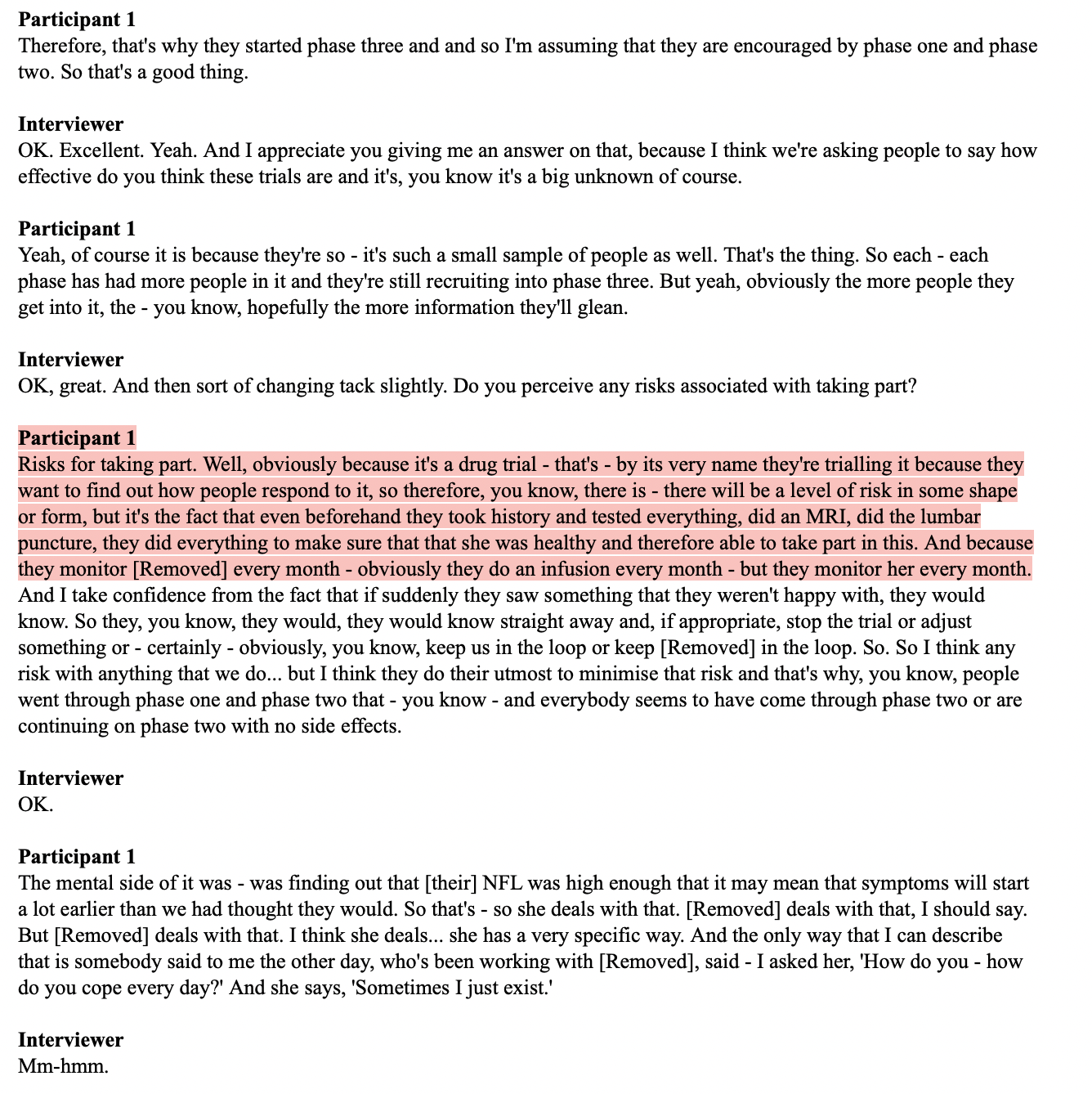


*Code: Impact upon mental health*


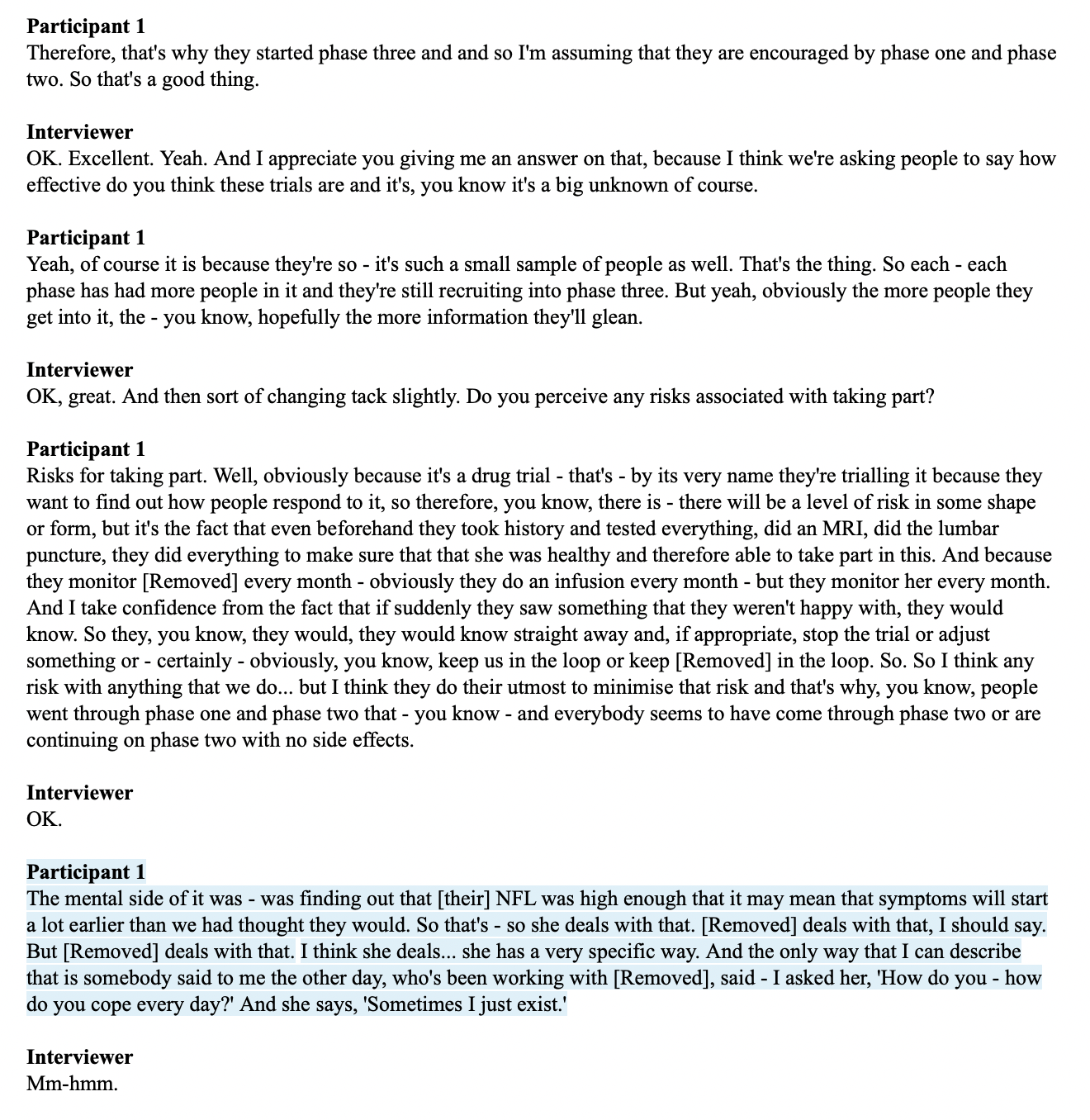


*Code: Trials provide hope*


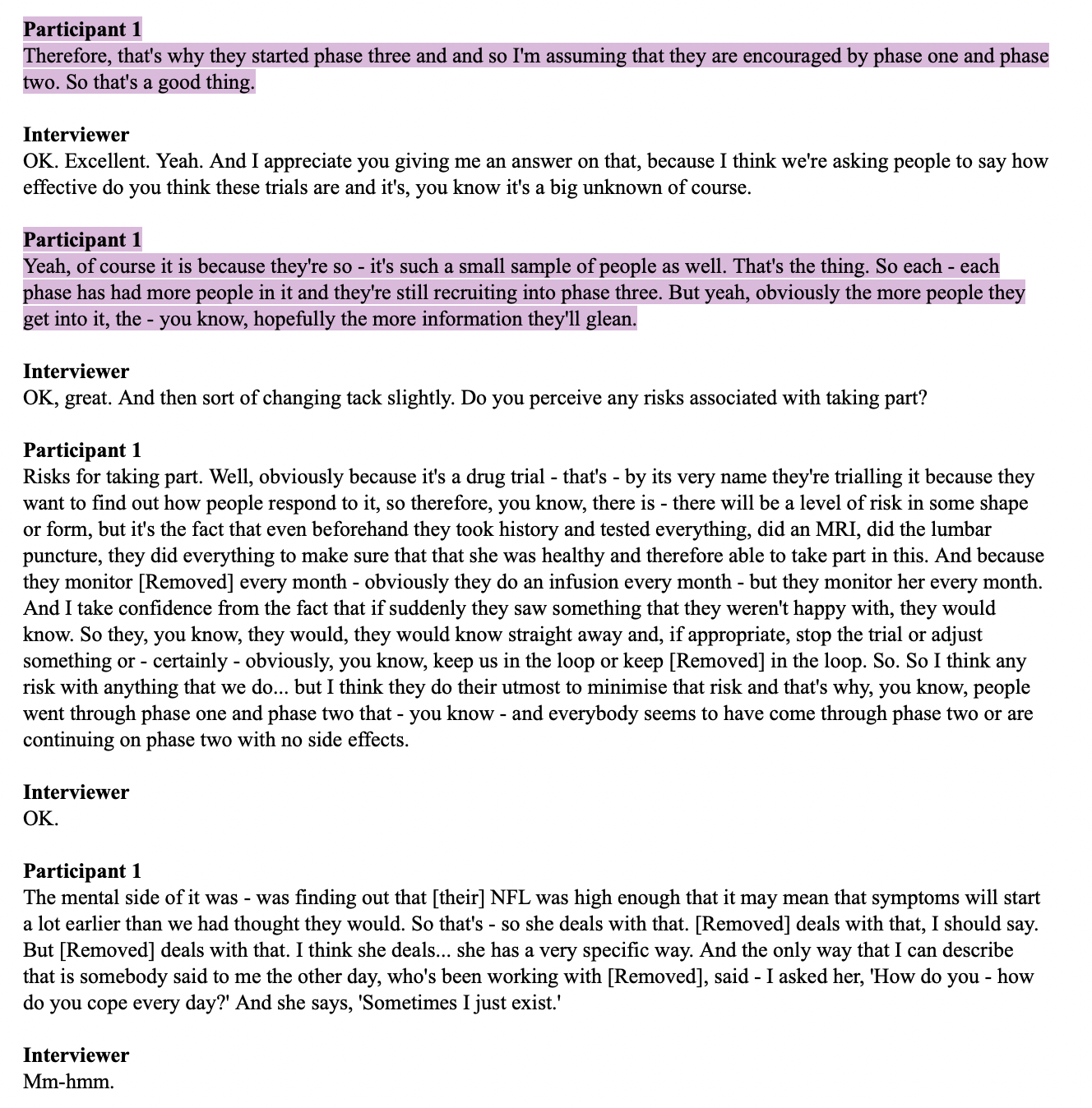


*Code: Impact of receiving new information regarding symptom onset*


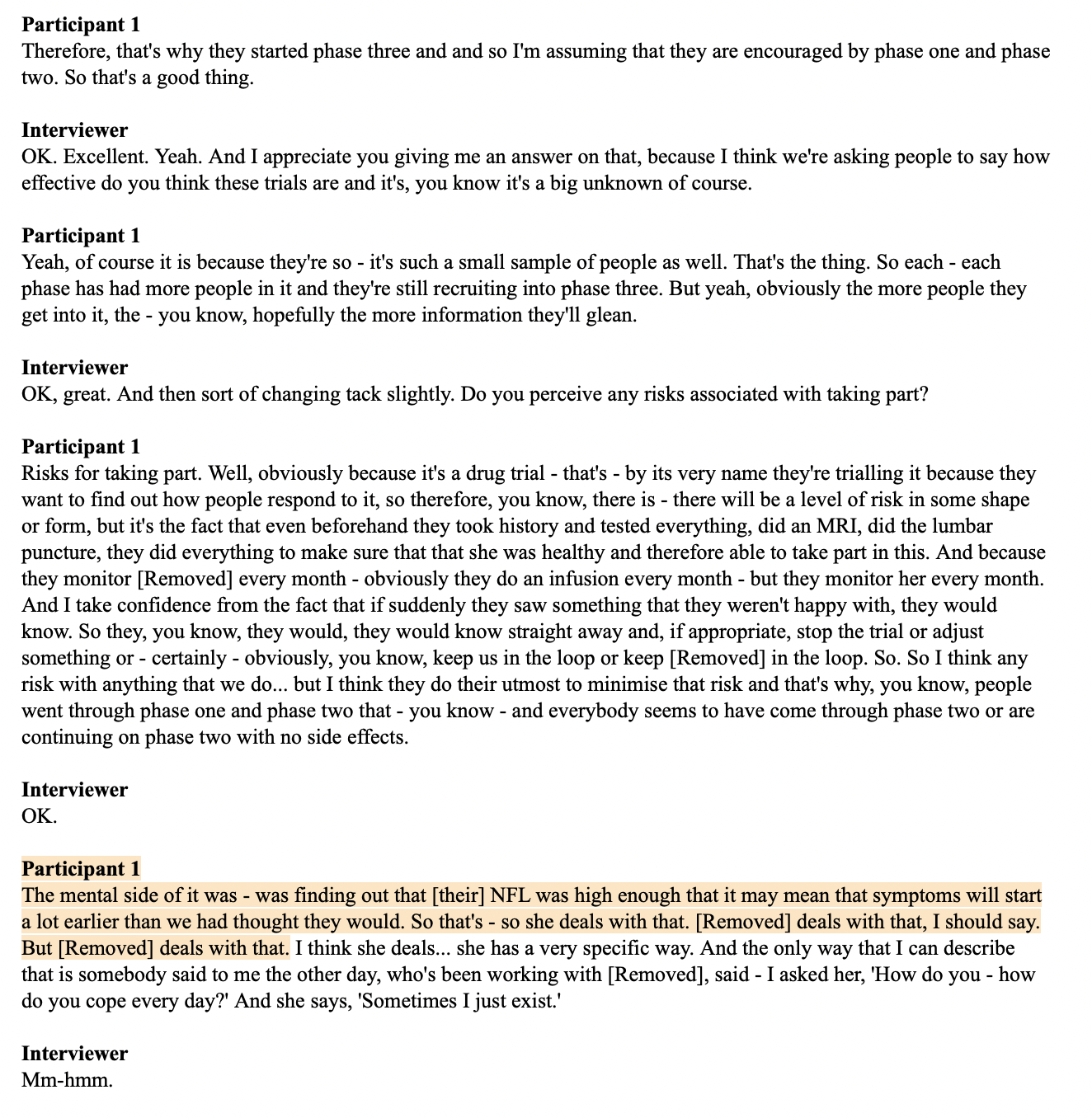


*Code: Faith in process and procedures*


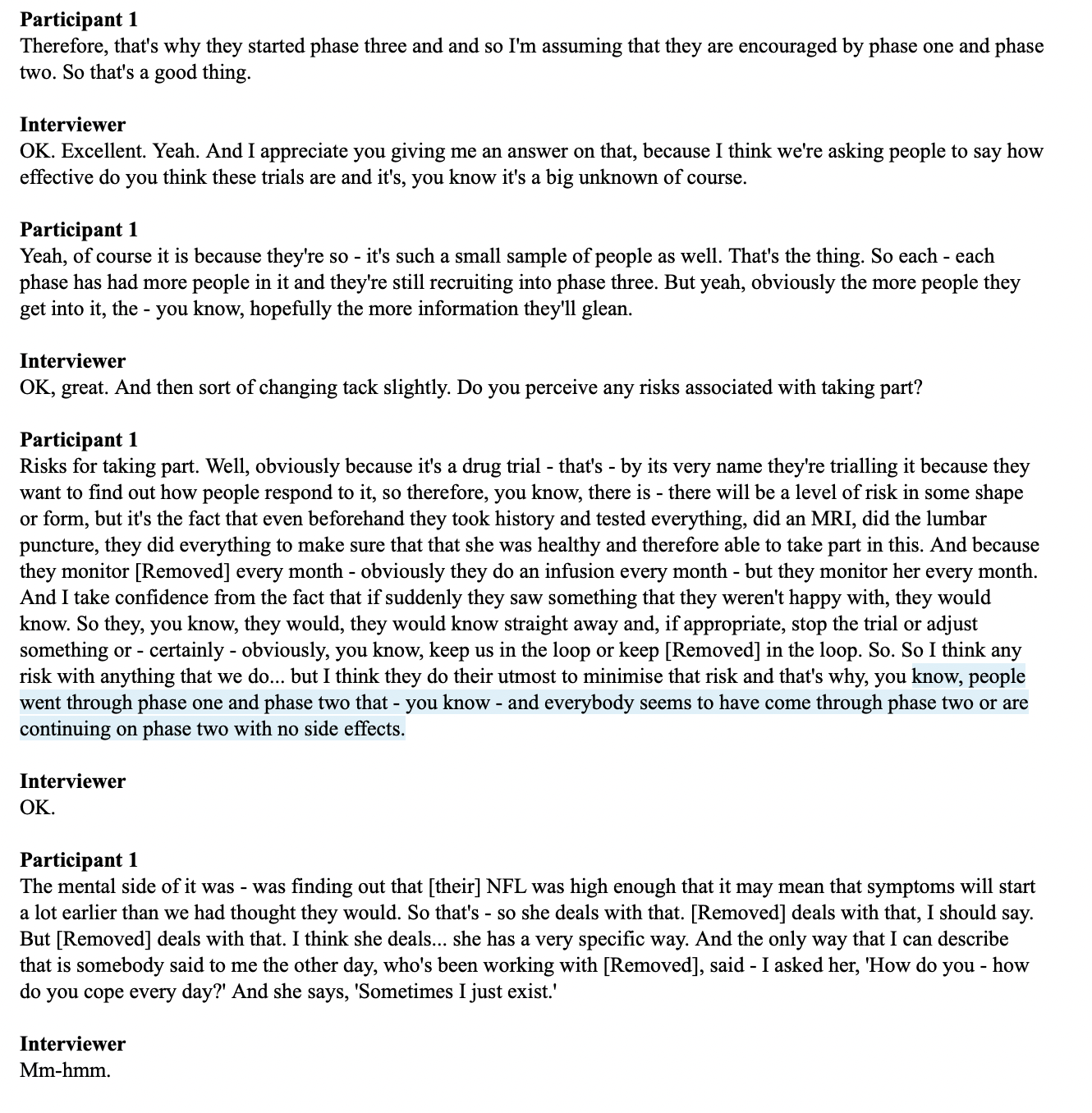


## Appendix C

### Example of Grouping Codes to Form a Sub-theme: Effects on Health and FTD Symptomatology

| **Code** | **Files** | **References** | **Theme** | **Sub-theme** |
| --- | --- | --- | --- | --- |
| Procedures - lumbar puncture | 11 | 24 | Effects on the Individual | Effects on Health and FTD Symptomatology |
| Unknown effects of new treatments | 10 | 28 | Effects on the Individual | Effects on Health and FTD Symptomatology |
| Physical impact | 9 | 28 | Effects on the Individual | Effects on Health and FTD Symptomatology |
| Impact of invasive procedures | 9 | 21 | Effects on the Individual | Effects on Health and FTD Symptomatology |
| Potentially slowing progression | 9 | 20 | Effects on the Individual | Effects on Health and FTD Symptomatology |
| Potentially finding a cure | 7 | 18 | Effects on the Individual | Effects on Health and FTD Symptomatology |
| Early access to treatment | 6 | 15 | Effects on the Individual | Effects on Health and FTD Symptomatology |
| Procedures - MRI | 6 | 12 | Effects on the Individual | Effects on Health and FTD Symptomatology |
| Benefiting directly | 6 | 10 | Effects on the Individual | Effects on Health and FTD Symptomatology |
| Access to better treatment | 5 | 7 | Effects on the Individual | Effects on Health and FTD Symptomatology |
| Procedures - gene therapy | 4 | 15 | Effects on the Individual | Effects on Health and FTD Symptomatology |
| Unknown effects of gene therapy | 4 | 12 | Effects on the Individual | Effects on Health and FTD Symptomatology |
| Procedures - blood tests | 4 | 7 | Effects on the Individual | Effects on Health and FTD Symptomatology |
| Trial potentially worsening symptoms | 4 | 5 | Effects on the Individual | Effects on Health and FTD Symptomatology |
| Procedures - intrathecal drugs | 3 | 7 | Effects on the Individual | Effects on Health and FTD Symptomatology |
| Risk of death | 3 | 3 | Effects on the Individual | Effects on Health and FTD Symptomatology |
| Access to experts and expertise | 2 | 9 | Effects on the Individual | Effects on Health and FTD Symptomatology |
| Perceived lack of improvements from trial | 2 | 3 | Effects on the Individual | Effects on Health and FTD Symptomatology |
| Access to funded treatment | 2 | 2 | Effects on the Individual | Effects on Health and FTD Symptomatology |
| Being monitored | 2 | 2 | Effects on the Individual | Effects on Health and FTD Symptomatology |
| Being part of something groundbreaking | 2 | 2 | Effects on the Individual | Effects on Health and FTD Symptomatology |
| Need to change current medication | 1 | 7 | Effects on the Individual | Effects on Health and FTD Symptomatology |
| Worry that side effects could be worse for older people | 1 | 3 | Effects on the Individual | Effects on Health and FTD Symptomatology |
| Potential for delirium | 1 | 2 | Effects on the Individual | Effects on Health and FTD Symptomatology |
| Hope for simulation of trials | 1 | 1 | Effects on the Individual | Effects on Health and FTD Symptomatology |
| Fear of missing chance of treatment | 1 | 1 | Effects on the Individual | Effects on Health and FTD Symptomatology |
| Reassuring nature of being part of an organisation | 1 | 1 | Effects on the Individual | Effects on Health and FTD Symptomatology |
| Worry that successful treatments may become expensive | 1 | 1 | Effects on the Individual | Effects on Health and FTD Symptomatology |
| Feeling of being a 'guinea pig' | 1 | 1 | Effects on the Individual | Effects on Health and FTD Symptomatology |
| Tiring nature of tests and assessments | 1 | 1 | Effects on the Individual | Effects on Health and FTD Symptomatology |
